# Mining, genetic mapping and expression analysis of EST-derived resistance gene homologs (RGHs) in cotton

**DOI:** 10.1186/s12870-014-0203-9

**Published:** 2014-07-27

**Authors:** Gaofeng Ren, Ximei Li, Zhongxu Lin

**Affiliations:** 1 National Key Laboratory of Crop Genetic Improvement, Huazhong Agricultural University, Wuhan 430070, China

**Keywords:** Cotton, PR, RGAs, Genetic mapping, Expression analysis

## Abstract

**Background:**

Cotton is the dominant textile crop and also serves as an important oil crop. An estimated 15% economic loss associated with cotton production in China has been caused by diseases, and no resistance genes have been cloned in this crop. Molecular markers developed from resistance gene homologues (RGHs) might be tightly linked with target genes and could be used for marker-assisted selection (MAS) or gene cloning.

**Results:**

To genetically map expressed RGHs, 100 potential pathogenesis-related proteins (PRPs) and 215 resistance gene analogs (RGAs) were identified in the cotton expressed sequence tag database, and 347 specific primers were developed. Meanwhile, 61 cotton genome-derived RGA markers and 24 resistance gene analog polymorphism (RGAP) markers from published papers were included to view their genomic distribution. As a result, 38 EST-derived and 17 genome-derived RGH markers were added to our interspecific genetic map. These 55 markers were distributed on 18 of the 26 cotton chromosomes, with 34 markers on 6 chromosomes (Chr03, Chr04, Chr11, Chr17, Chr19 and Chr26). Homologous RGHs tended to be clustered; RGH clusters appeared on 9 chromosomes, with larger clusters on Chr03, Chr04 and Chr19, which suggests that RGH clusters are widely distributed in the cotton genome. Expression analysis showed that 19 RGHs were significantly altered after inoculation with the V991 stain of *Verticillium dahliae*. Comparative mapping showed that four RGH markers were linked with mapped loci for Verticillium wilt resistance.

**Conclusions:**

The genetic mapping of RGHs confirmed their clustering in cotton genome. Expression analysis and comparative mapping suggest that EST-derived RGHs participate in cotton resistance. RGH markers are seemed to be useful tools to detected resistance loci and identify candidate resistance genes in cotton.

## Background

Cotton is the leading textile crop and also serves as an important oil crop worldwide. Cotton is also widely used for studies of disease resistance [1]–[3]. The genus *Gossypium* contains approximately 50 species; *G. hirsutum* and *G. barbadense* are the two most extensively cultivated tetraploid species. *G. hirsutum* accounts for approximately 95% of the total cotton production due to its high yield and ability to adapt to various environmental conditions, although its disease resistance is weaker than that of *G. barbadense*. Previous studies showed that four different type of *G. barbadense* were resistant to both severe-virulence strains and intermediate-virulence stains of the fungus [4]. These two species are commonly used in cotton resistance studies [5]–[7].

From sowing to harvest, cotton may suffer from more than 40 types of diseases, which lead to economic losses of approximately 15% in China. The most serious of these diseases are Verticillium wilt and Fusarium wilt [1]. These losses can be decreased as the diseases can be effectively controlled through screening resistant germplasms or breeding transgenic resistant cultivars; however, molecular resistance breeding is difficult in cotton because no resistance genes have been cloned and few molecular markers that are tightly linked with target genes or quantitative trait loci (QTL) have been found [6],[8]–[15].

These difficulties can be overcome via the development of resistance gene homologue (RGH) markers. The broad definition of RGHs includes defense gene analogs (DGAs) and resistance gene analogs (RGAs). Pathogenesis-related (PR) proteins are defense genes and have some biological properties such as glucanase, chitinase and peroxidase activities, which contribute to the development of resistance to various fungal diseases [16]. The products of most R genes contain some similar conserved domains such as nucleotide binding sites (NBSs), leucine-rich repeats (LRRs), transmembrane domains (TMs) and Toll-interleukin-1 regions (TIR). The mechanisms of action of most R genes, including *Pto*, *N*, *L6*, *RPS 2*, *Cf-9* and *Xa21,* are similar those of R genes encoding proteins that can react with *avr* gene products and ultimately activate defense responses [17].

RGH markers might be part of the resistance gene or pseudogene and tend to form clusters, which makes them much more effective than random markers in map-based cloning and marker-assisted selection (MAS) [18]. For example, two RGA markers that co-segregated with the powdery mildew resistance locus *Run*1 were detected in grapevine [19], and the *Yr5* gene for resistance to wheat stripe rust was found to co-segregate with two RGAP markers [20].

Degenerate primers designed based on conserved motifs of known resistance proteins have been widely used to amplify RGHs. Many RGHs have been cloned from the cotton genome with degenerate primers. Thirty-one NBS-type RGAs were detected in tetraploid sea-island cotton [5]. Seventy-nine NBS sequences, 21 serine/threonine kinase (STK) sequences and 11 DGAs were cloned with 13 degenerate primers [21]. However, few RGHs have been mapped. Sixteen NBS-LRR-type RGA markers have been localized, 9 RGA markers were located on two homologous chromosomes (Chr12 and Chr26), and 212 RGA and RGA-AFLP markers were located on 18 chromosomes using 14 primer pairs [7],[22],[23]. Data mining from the cotton genome database was also used to detect resistance genes, and 355 NBS-encoding resistance genes were identified from the diploid cotton species *G. raimondii*[3].

In this study, we focused on identifying potentially expressed RGHs by searching the cotton EST database, and we designed corresponding primers to map these RGHs to characterize their genomic distribution. EST-derived RGHs are preferable to genome-derived RGHs because there is typically no interference from pseudogenes, which makes EST-derived RGHs more likely to be functional genes. Moreover, EST-derived RGH markers co-segregating with resistance genes may be the R genes themselves, which makes them more effective in MAS and map-based cloning from a complex genome. Expression analyses were also performed to determine whether these RGHs were involved in the resistance response.

## Methods

### Plant materials and RNA extraction

The mapping population used to map the RGHs is the interspecific BC_1_ population derived from [(Emian22 × 3-79) × Emian22], which includes 141 individuals and 2316 markers [24]. Seedlings of the mapping parents (*G. hirsutum* cv. Emian22 and *G. barbadense* acc. 3–79) with two euphyllas were inoculated with the V991 strain of *V. dahliae* by root dipping for 1 minute. The roots were collected at 0, 6, 24, 48, 72 and 96 hours after inoculation, and total RNA was extracted using the guanidine thiocyanate method [25]. First strand cDNAs were synthesized using 4 μg of RNA from each sample following the instructions provided with the Superscript® III RT kit (Invitrogen, Carlsbad, USA).

### Mining of EST-derived RGHs and primer design

The EST database of *G. hirsutum* (release 3) was downloaded from the Institute for Genomic Research [26]. Because the sequences in this database were unique and had been annotated, three key words (“PR”, “Pathogenesis” and “Pathogenesis-related protein”) were used to search this database to obtain PR sequences directly [27]. Non-targeted sequences and redundancies were manually excluded. Target sequences were submitted to Primer-BLAST [28], and specific primers were designed with the following criteria: optimum annealing temperature 55°C and PCR product size 100–500 bp. The primers were named using the sequence ID + PR.

Three cotton EST databases (*G. arboreum* release 3, *G. raimondii* release 2 and *G. hirsutum* release 3) were downloaded from TIGR to obtain RGAs [26]; 196 different types of resistance proteins from *Arabidopsis thaliana* and 95 R proteins from different species were collected [29]. The program tBLASTn was utilized to align the collected R proteins against the EST sequences in the three databases, employing an E-value of 10^−10^. EST sequences showing ≥40% identity to R proteins were selected, and these selected nucleic acid sequences were translated to protein sequences using the three forward frames as well as the three reverse frames for convenience. Conserved Domain Search which was provided by the National Center for Biotechnology Information (NCBI) was performed to identify the conserved protein domains on translated proteins [30]. EST sequences were classified as RGAs if their protein sequences contained resistance-related domains, such as NBS, LRR, TIR and Hs1pro-1.

RGA primers were designed to ensure that specific regions were amplified. To improve the primer specificity, we attempted to design the primer pairs such that they surrounded the conserved domains. Occasionally, two or more primers were designed based on one sequence that contained more than one conserved domain. The primers were named using the sequence ID + RGA. We found that some RGAs contained potential introns by aligning the RGA sequences with the *Arabidopsis thaliana* genome. Some primers were designed surrounding these introns to increase the level of polymorphism; these primers were named using the sequence ID + RGA-ILP. The other criteria were the same as those used for PR primer design.

### RGA primers collected from the literature

A total of 61 specific primers that were designed based on upland cotton RGAs and 24 RGAP primers that were previously used to amplify RGAs from different plant species were included in this study [23],[31]. These 61 primers were designed based on 61 upland cotton consensus sequences or individual RGA sequences that were amplified with degenerate primers. RGAP primers were designed based on the conserved motifs of known plant R genes. These 85 primers were used to genotype the mapping population directly.

### RGH marker analysis and linkage map construction

All the 347 EST-derived primers, 61 genome-derived RGA primers and 24 RGAP primers were used to genotype *G. hirsutum* cv. Emian22 and *G. barbadense* acc. 3–79 for screening polymorphic markers. Then, the polymorphic markers were used to genotype the 141 individuals of the BC_1_ population.

PCR reactions were performed in 10 μL volumes containing 25 ng of genomic DNA, 0.4 μM each primer, 2.0 mM MgCl_2_, 0.25 mM dNTPs and 0.8 U of *Taq* DNA polymerase. The PCR reaction conditions were 5 min at 94°C, followed by 35 cycles of denaturation at 94°C for 50 s, annealing at 56°C for 45 s and extension at 72°C for 1 min. A final extension was performed at 72°C for 5 min. The PCR products were separated on an 8% non-denaturing polyacrylamide gel with a constant power of 15 W for 4.5 h to detect polymorphisms (Single strand conformation polymorphism, SSCP) [32]. The PCR reaction conditions for the published primers were the same as those described in the original references but genotyped by SSCP to detect more polymorphism.

Primers that were polymorphic between the mapping parents were used to genotype the 141 individuals of the BC_1_ population. RGH markers were added to the genetic map using the program JoinMap 3.0 [33]. The logarithm of odds (LOD) threshold was 5.0, and the maximum recombination rate was 0.4. Map distances in centiMorgans (cM) were calculated using the Kosambi mapping function [34]. The resulting linkage map was drawn using MapChart 2.2 software [35].

### Expression analyses by semi-quantitative RT-PCR (SQ-PCR) and qRT-PCR

In addition to the EST-derived RGH primers, the 61 genome-derived specific primers collected from the literature were also used in SQ-PCR to detect differences in expression between Emian22 and 3–79 after inoculation with *V. dahlia*; UB7 was used as an internal control gene. The cDNAs of Emian22 and 3–79 that were obtained 0, 6, 24, 48, 72 and 96 hours after inoculation were used as templates, and the SQ-PCR reactions were the same as those described in the section “RGH marker analysis and linkage map construction” except that the cycle number was reduced to 28 and the amplified products were separated on a 1% agarose gel to detect the expression difference.

qRT-PCR analyses were carried out using RGH primers that showed differential expression during SQ-PCR. The qRT-PCR protocol was the same as that described by Munis et al. [2]. UB7 was used as the internal control gene, and error bars indicate the standard deviation (SD) of three replicate samples.

## Results

### Sequence mining and primer design

A total of 100 PR sequences were identified from the *G. hirsutum* EST database, and most of these sequences belonged to the PR-10 class. In fact, several PR sequences were also identified from other cotton species; however, they were highly homologous to those from *G. hirsutum* and were therefore excluded from this study (data not shown). Sequence-specific primers were designed for each sequence using Primer-BLAST (Additional file 1: Table S1). From the three cotton EST databases, a total of 215 unique sequences were classified as RGAs by tBLASTn and a conserved domain search. Finally, 247 primers were designed (Additional file 2: Table S2). The 215 RGAs contained different types of conserved domains; LRRNT/LRR, NB-ARC and PKc/PKc_like were the three most frequent domains in RGAs (Table 1).

**Table 1:** Subgroup numbers and origins of EST-derived RGHs

**EST-RGH types**	**Conserved domain**^**a**^	***G. arboreum***	***G. raimondii***	***G. hirsutum***	**Total**
RGA	LRRNT/LRR	10	15	40	65
	NB-ARC	4	11	20	35
	PKc/PKc_like	2	14	16	32
	PLN00113 ^b^	2	7	18	27
	Mlo	2	8	6	16
	TIR	2	\	11	13
	Hs1^pro-1^	1	4	6	11
	CHORD	5	1	2	8
	Other	\	1	7	8
PR	\	\	\	100	100
Total number	\	28	61	226	315
Number of mapped loci	\	2	4	11/21^c^	38

^a^Some RGA contained more than one domain; however, only the domain that obtained the highest score during the conserved domain search was counted.

^b^Leucine-rich repeat receptor-like protein kinase.

^c^Eleven and 21 mapped loci belong to RGA and PR, respectively.

### Genetic mapping of RGHs

The mapping parents *G. hirsutum* cv. Emian22 and *G. barbadense* acc. 3–79 were used to screen polymorphic primers, which were subsequently used to genotype the 141 individuals of the BC_1_ population. Although the genetic distance between adjacent RGH loci was not always small, RGH loci tended to occur in a relatively small region. Considering the small number of RGH markers, we defined a cluster with a general concept that two or more RGHs markers occurring within 10 cM of each other.

**Figure 1:**
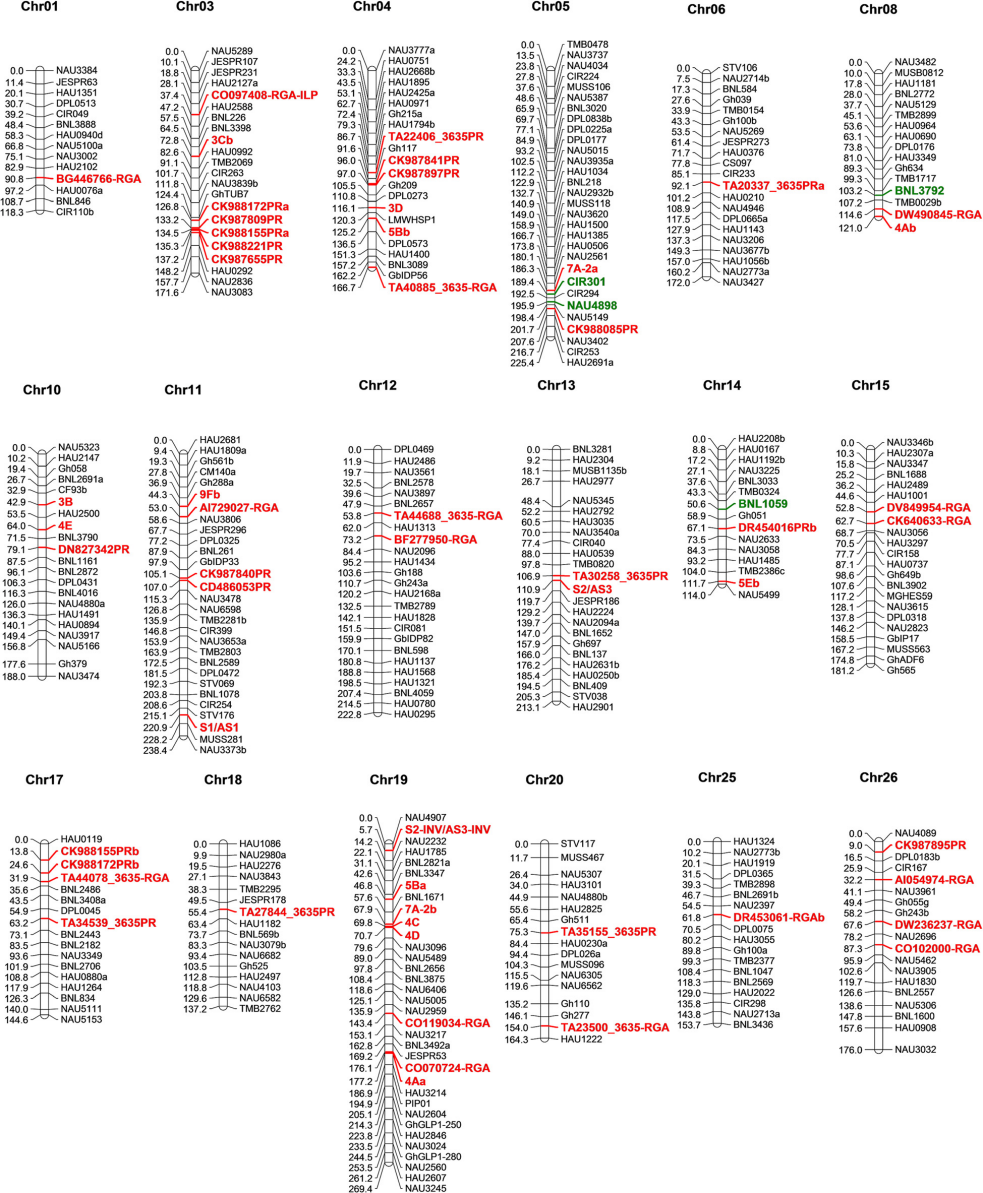
**The distribution of RGH loci on the interspecific linkage map.** RGH loci are shown in bold, red font. Markers associated with Verticillium wilt resistance are shown in bold, green font. For simplicity, only subsets of the markers on the original map are shown on this map to maintain the gaps at approximately 10 cM.

Of the 100 PR primers, 72 were successfully amplified with genomic DNA and 25 were polymorphic; 21 PR loci were added to the genetic map, and CK988155PR and CK988172 each contributed two loci (Figure 1). These 21 PR loci were distributed on 12 chromosomes, and six clusters consisting of two to five PR loci were identified. Five PR loci (CK988172PRa, CK987809PR, CK988155PRa, CK988221PR and CK987655PR) clustered on Chr03, and four of them were closely distributed. The corresponding nucleotide sequences of any two of these five sequences shared ≥90% similarity. CK987841PR and CK987897PR were closely linked on Chr04, whereas CK987840PR and CD486053PR were located on Chr11 with a genetic distance of 1.9 cM.

Of the 247 RGA primers, 196 were successfully amplified. However, only 19 were found to be polymorphic, and 17 RGA loci were added to the genetic map (Figure 1). These 17 loci were distributed on twelve chromosomes, each of which contained 1 to 3 RGA loci. DV849954-RGA and CK640633-RGA were located on Chr15, and their genetic distance was 9.9 cM.

Fifty-five of the 61 published genomic DNA-derived RGA primers were successfully amplified, and 14 loci were added to the genetic map (Figure 1). These 14 RGA loci were distributed on 8 chromosomes, and Chr19 contained 5 of the 14 loci. The corresponding nucleotide sequences of 3 closely linked RGA loci (7A-2b, 4C and 4D) on Chr19 shared ≥80% identity when any two of these three sequences were compared. Three loci derived from 24 RGAP primers (S1/AS1, S2/AS3 and S2-INV/AS3-INV) were located on Chr11, Chr13 and Chr19, respectively.

We found that the 55 RGH loci were not equally distributed throughout the cotton genome. Five chromosomes (Chr03, Chr04, Chr11, Chr17 and Chr19) contained 7, 6, 6, 4 and 8 RGH loci, respectively, while another 8 chromosomes contained zero loci and the remaining 13 chromosomes contained 1 to 3 RGH loci. Furthermore, more than half of the RGH markers were members of a cluster, and the distance between adjacent RGH loci was no more than 10 cM. RGH clusters were abundant in the cotton genome; clusters were found on 11 chromosomes, and the three biggest clusters were located on Chr19, Chr03 and Chr04.

### In *silico* mapping of RGH markers for Verticillium wilt resistance

Many single markers and QTLs associated with Verticillium wilt resistance have been reported in cotton [6],[8],[10]–[15]. By in *silico* comparative mapping, we found that some RGH markers were linked with Verticillium wilt resistance-related markers (Table 2). Three SSR markers (CIR301, BNL1059 and BNL3792) linked with Verticillium wilt resistance QTL also appeared in our BC_1_ genetic map and had the same chromosomal locations. The genetic distance between these 3 markers and the nearest RGH markers (7A-2a, DR454016PRb and DW490845RGA) were 3.0 cM, 16.4 cM and 11.3 cM, respectively [11].

**Table 2:** Locations of Verticillium wilt resistance-related markers and adjacent RGH markers

**SSR marker**^**a**^	**RGH marker**^**b**^	**Genetic distance**	**Chromosome**	**Reference**
CIR301	7A-2a	3.0 cM	Chr05	[11]
BNL1059	DR454016PRb	16.4 cM	Chr14	[11]
BNL3792	DW490845RGA	11.3 cM	Chr08	[11]
NAU4898	CK988085PR	5.8 cM	Chr05	[13]

^a^SSR markers related to Verticillium wilt resistance.

^b^RGH markers linked with Verticillium wilt resistance-related SSR markers.

### Expression analysis

Among the 408 primers which were analyzed by SQ-PCR to determine whether they were expressed after inoculation with *V. dahliae*, 64 PR primers, 86 RGA primers and 11 genomic DNA-derived RGA markers from the literature were found to be expressed (Additional file 1: Table S1, Additional file 2: Table S2 and Additional file 3: Table S3). Up to 64% of the PR primers and 35% of the RGA primers, which were derived from EST sequences, were found to be expressed, while this proportion was 18% for the genomic DNA-derived RGA markers.

**Figure 2:**
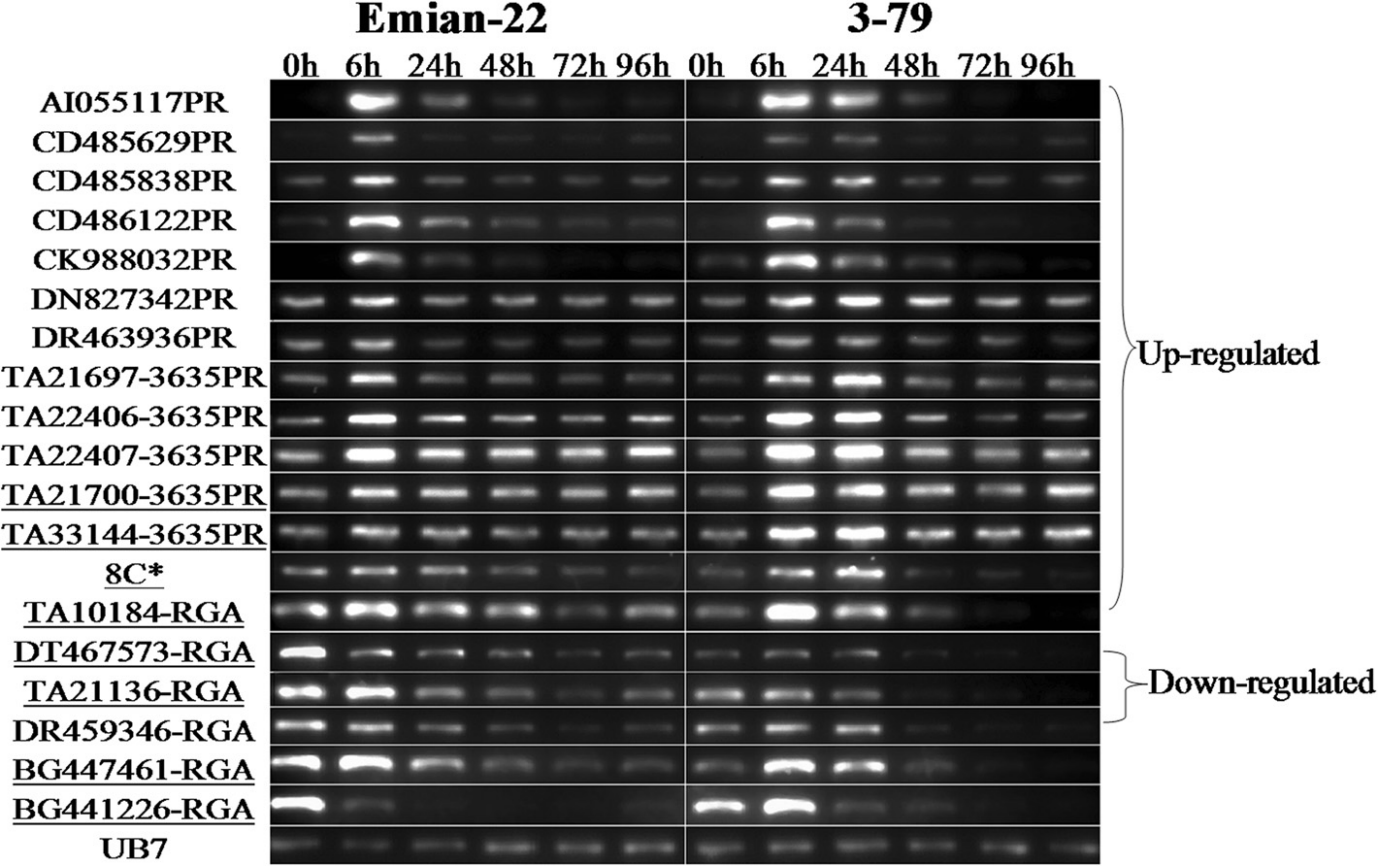
**SQ-PCR expression analysis of RGHs.** UB7 is a constitutively expressed gene in cotton and was used as an internal control gene. “0-96 h” indicates 0–96 h after inoculation. Markers showing differences between Emian22 and 3–79 are underlined.

**Figure 3:**
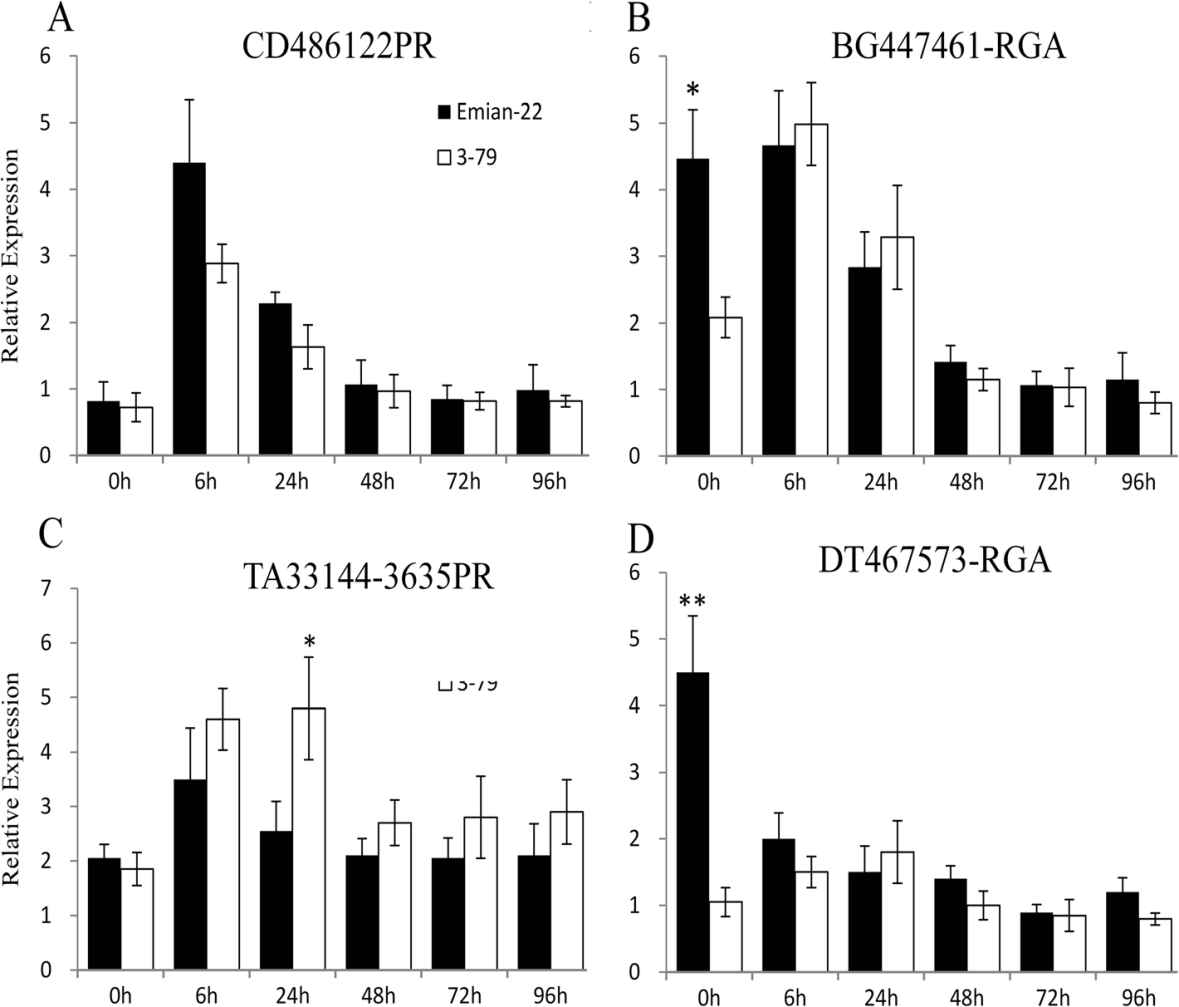
**qRT-PCR analysis of four RGHs.** The expression levels of these four RGHs were altered after inoculation and were significantly different between Emian22 and 3–79. UB7 is a constitutively expressed gene in cotton and was used as the internal control gene, and error bars indicate the SD of three sample replicates (t-test, *P < 0.05, **P < 0.01). “0-96 h” indicates 0–96 h after inoculation. Markers showing differences between Emian22 and 3–79 are underlined.

For most of the expressed RGH primers (approximately 88.2%), the expression levels were not different between Emian22 and 3–79 at 0, 6, 24, 48, 72 or 96 hours after inoculation. The remaining 12 PRs, 6 RGAs and 1 genomic DNA-derived RGA were found to be differentially expressed by SQ-PCR (Figure 2). TA10184-RGA, 8C* and all 12 PRs were upregulated in the seedlings of the two parents, although the expression levels of four markers (TA21700-3635PR, TA33144-3635PR, TA10184-RGA and 8C*) were much higher in 3–79. DT467573-RGA, TA21136-RGA and DR459346-RGA were downregulated; however, the initial expression levels of the first two markers were higher in Emian22. DR447461-RGA and BG441226-RGA were downregulated in Emian22 but upregulated in 3–79. Similar results were obtained by qRT-PCR. Additionally, the expression level of some RGHs was significantly different between Emian22 and 3–79 (Figure 3). The expression levels of BG447461-RGA and DT467573 in Emian22 at 6 h were significantly higher than those in 3–79, while the expression level of TA33144-3635PR was significantly higher in 3–79 at 24 h (Figure 3).

## Discussion

### The advantage of mining RGHs from an EST database

Most cotton RGHs were derived from genomic DNA by degenerate primer amplification or data mining, and they rarely corresponded with functional genes due to the interference of large numbers of pseudogenes. When RGHs were amplified from cDNA using degenerate primers, lowly expressed RGHs were easily lost due to the interference of highly expressed RGHs in random cloning. EST database mining can overcome these disadvantages, as the target sequences are more likely to be functional genes and the large amount of data could dampen the effect of expression level. The proportion of EST-derived RGHs was much higher than that of genome-derived RGHs, which suggests that EST-derived primers were more likely to be functional.

The RGHs obtained through EST database mining were more diverse. Most of the degenerate primers were designed based on a limited number of motifs, which meant that only specific parts of the RGHs could be amplified. While mining RGHs from the EST database, R genes and defense genes that were not suitable for degenerate primer design could be used to detect RGHs. RGHs that contained conserved domains could be detected. In this study, PR sequences included different classes, such as PR-10, PR-2, PR-5 and PR-7. The 215 RGAs contained different resistance-related domains, and some of them were not likely to appear through the use of degenerate primers. LRR is the most frequently observed domain because it appears in many types of R genes. TIR, NB-ARC and PKc are found in common R genes. Mlo is related to resistance to powdery mildew fungus and dysregulated cell death control [36]. Plant Hs1pro-1 proteins are believed to confer nematode resistance [37]. CHORD is required for disease resistance signaling in barley [38].

### The low polymorphism of RGA primers

Of the196 successfully amplified RGA primers, only 19 were found to be polymorphic by SSCP which can detect single base mutation. In fact, the polymorphism level was even lower with denaturing polyacrylamide gel and high power electrophoresis which can only detected fragment length polymorphism.

This might be caused the primer design strategies. In our study, in order to make sure that we target RGAs, the primers were designed to target the conserved protein domains, which resulted in that the nucleic acid sequences were relatively conservative.

### The distribution of RGHs in the cotton genome

A total of 55 markers were added to the genetic map, and the distribution that we observed was not identical to what was observed in other studies in cotton. Sixteen NBS-LRR-type RGA markers were mapped, and 12 of them were located in the A_T_ genome [22]. However, in our study, only 1 out of 5 NBS-LRR type markers was located in the A_T_ genome, and all 55 markers were basically evenly distributed in the A_T_ and D_T_ genomes. In cotton diploids, NBS domain-encoding genes of D_5_ genome of *G. raimondii* were expansded after its divergence from A_2_ genome of *G. arboretum*[39]. Thus, we could not conclude with certainty that RGHs were more abundant in the A_T_ or D_T_ genome in tetraploid cotton.

It is known that most RGHs exist in the form of clusters, regardless of whether they are placed on a physical map or a genetic map [18],[40],[41]. RGA clusters were found on Chr23 and Chr17 [22]. Two RGA clusters were mapped on the 2 homologous chromosomes of Chr12 and Chr26 [23]. Clusters comprising RGA and RGA-AFLP markers were found on Chr6, Chr12 and Chr15 [7]. Forty-nine NBS-type RGA clusters were located on the physical map of the D_5_ genome of *G. raimondii*[3]. We also found that RGH clusters were located on 11 chromosomes, with larger clusters on Chr03, Chr04 and Chr19, which indicated that the distribution of EST-derived RGHs was the same as that of genome-derived RGHs.

Two clusters consisting of highly homologous sequences were found on Chr03 and Chr19. These homologous sequences might be derived from tandem duplication and recombination events, which have been important mechanisms of R gene evolution.

Three RGH primers contributed two loci each, and loci derived from the same primer were located on the homologous chromosomes. The loci 7A-2a and 7A-2b were located on homologous chromosomes 05 and 19. CK988172PRa and CK988172PRb were located on homologous chromosomes 03 and 17, and CK988155PRa and CK988155PRb were also located on homologous chromosomes 03 and 17. Interestingly, the distance between CK988172PRa and CK988155PRa was similar to the distance between CK988172PRb and CK988155PRb. The similar locations of these homologous markers might be caused by concerted evolution, which is an important evolution mechanism of rDNA in heterologous tetraploid plants. Another explanation was the collinear of these two subgenomes, because most chromosomes of *G. raimondii* and *G. arboretum*, the ancestors of tetraploid cotton, were highly collinear [39].

### Expression analysis

Nineteen RGHs might be involved in cotton Verticillium wilt resistance because their expression levels were significantly changed after inoculation (Figure 2). PR genes are usually related with plant broad-spectrum resistance, and as many as 12% of the PR sequences used in this study were found to be differentially expressed. All 12 PR sequences were upregulated after inoculation, which was in agreement with the biological properties of PR proteins. Most PR proteins had the actives of glucanase, chitinase, peroxidase and so on. Upregulated express of PR will promoted the synthesis of PR proteins, and improve the resistance to various fungal diseases [16]. Additionally, the generation and accumulation of PR proteins is one of the most important mechanisms in the plant resistance response. Seven RGAs (2.27%) were differentially expressed, which suggests that RGAs are more specific than PR sequences in the plant resistance response. TA10184-RGA, BG447461-RGA and BG441226-RGA were homology with leaf mould resistance gene *Cf-5* and were upregulated in *G. barbadense* acc. 3–79; the upregulated of expression was conducive to react with *avr* gene products and ultimately activate defense responses.

The expression patterns of TA21700-3635PR, TA33144-3635PR and the 7 RGAs were different between the seedlings of these two parents. This result might be due to the fact that the resistance of *G. barbadense* was stronger than that of *G. hirsutum*. The potential functions of these sequences as potential resistance-related genes should be verified by transgenic experiments. Additionally, these sequences could be used for the identification of resistance loci.

Three RGH loci (7A-2a, DR454016PRb and DW4907845RGA) were found to be linked with resistance-related SSR markers. Considering that most RGHs were found in clusters and approximately 81% of NBS-type RGA loci were located in clusters, these three loci may exist in clusters, and some members of these clusters might be target genes that contribute to the resistance to Verticillium wilt [3]. DR454016PRb and DW490845RGA did not yield PCR products when SQ-PCR experiments were performed; thus, they might be not induced by the V991 strain of *Verticillium* wilt but could be induced by other pathogen types. Locus 7A-2a was expressed and the corresponding nucleotide sequence showed high homology to DNA-binding proteins; thus, it might be involved in signal transduction during the resistance response.

## Conclusions

In this study, we found that the expression levels of some RGHs were significantly changed after inoculated, and some RGH markers were linked with resistance-related markers. These differentially expressed RGHs could be used to identify resistance related genes by transgenes, and RGH markers are seem to be useful tools to detect resistance loci in cotton. In this study, we demonstrate the effectiveness of RGH and it might accelerate the process of breeding resistance cotton cultivars.

## Competing interests

The authors declare that they have no competing interests.

## Authors’ contributions

ZL conceived and designed the study. GR performed experiments and wrote the manuscript. XL assisted with experiment design and discussed analysis. All authors read and approved the final manuscript.

## Additional files

## Supplementary Material

Additional file 1: Table S1. The sequences of PR-type primers. The sequences and detail information of PR primers. Click here for file

Additional file 2: Table S2. The sequences of RGA primers. The sequences and detail information of RGA primers. Click here for file

Additional file 3: Table S3. Genomic DNA-derived RGA markers. The sequences and detail information of genomic DNA-RGA primers. Click here for file
